# A Mini Review of Using the Oralift Appliance and a Pilot Study to See if 3D Imaging Techniques Could Improve Outcomes

**DOI:** 10.2174/1874210601812010283

**Published:** 2018-03-30

**Authors:** N. K. Mohindra

**Affiliations:** Oralift Ltd, 18 Wimpole Street, London W1G 8GD, London, UK

**Keywords:** Oralift appliance, 3D imaging techniques, 2D photography, Temporomandibular disorder, Thermoplastic material, Tick test

## Abstract

**Background:**

Occlusal appliances of various designs have been described in the literature. They usually have to be worn for substantial periods including night time to have the desired effect for which they are designed. The Oralift^®^ appliance has been designed to address the signs of facial ageing and to help stop parafunctional habits. The appliance is based on the principles of the pivot appliance and is worn for very short periods, never at night and not even every day. The maximum usage recommended is two hours every third day.

**Objective:**

This is a review of five patients who have been treated with Oralift^®^ with the aim of assessing whether the visual changes seen by wearing the appliance can be quantified by changes in volume as measured by 3D Imaging, and if this quantification could be useful in improving the outcomes for each patient.

**Methods:**

The patients were fitted with the appliances in general practice, and 2D images were taken before, during and after treatment. Afterwards, the patient attended King’s College London, to have 3D imaging.

**Results:**

3D imaging has been proved far superior to standardized 2D photography in assessing the changes taking place on the face, and helped quantify the volume changes.

**Conclusion:**

To further improve the outcome for each patient, the 3D imaging should be done before the visit to the practitioner or ideally by the practitioner so that the results could be assessed, and the treatment adjusted accordingly. The implication of the volume changes requires a much larger study.

## 1 INTRODUCTION

**Fig. (1):**
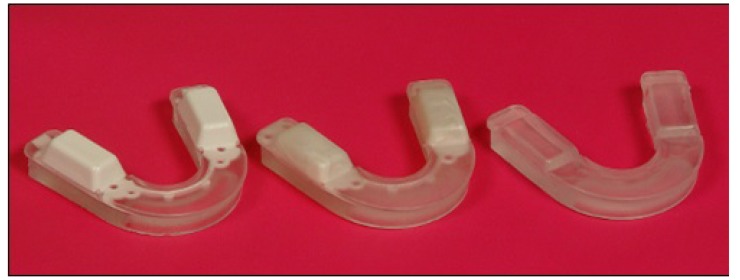
Oralift appliances with 3mm, 5mm and 7mm pivots with a thermoplastic base.

In this study the aim was to see if 3D imaging could quantify the changes taking place in patients using the Oralift^®^ appliance and whether this could help improve the outcomes for the patients. The Oralift^®^ is composed of a thermoplastic material that moulds on to the mandibular teeth and has two non-deformable pivots which sit on the premolar and molar teeth. The height of the pivots varies from 3 mm-7 mm (Fig. **1**).

Appliances have been used in dentistry for a number of purposes. Temporomandibular disorders have been treated with occlusal splints since the late 19th century [1]. It was not until the 1940’s that Costen’s [2] concept of altering patients’ occlusions with splint therapy came into fashion. In the early 20 ^th^ century, functional appliances were started to be used in orthodontics [3]. The use of removable appliances in comparison to fixed appliances has declined over the recent years [4]. Nowadays, appliances are used not only for TMD and orthodontics, but for sleep apnea and to help stop bruxism [5-7]. There have been soft and hard appliances which give partial or full coverage [8]. All the appliances rely on two principles: 1) facial muscles take time to adapt and 2) teeth have to make contact with the appliance. Some have facets on the appliance which have to provide guidance to the jaw in movement, or help the jaw to reposition itself into a more favourable position. The Oralift^®^ appliance is designed to be worn for very short periods and the aim is not to allow the opposing teeth to make contact with the appliance. This will help the facial muscles to adapt and allow an increase in the freeway space.

The Oralift^®^ appliance is based on the principles of the pivot appliance first used by Sayers [9] and later by Watt [10]. This appliance has been designed to improve the signs of facial ageing. Facial ageing is a multi-dimensional, multi factorial process [11, 12]. The process of ageing transforms the face with changes that are usually classified as either chronological or photo induced. These changes affect the shape, the texture and the colour of the face. The shape of the face is transformed by changes in the bones and the soft tissues (muscles, fat and skin). Gravity also plays a part. Skin texture is affected by the degradation of facial collagen. This leads to the atrophy of the skin layers and loss of elastosis [13, 14]. The changes to the ageing face can have a profound effect on self-perception. The improvement of self-image is an important step towards happiness for many patients.

The aging face has traditionally been treated by a number of specialists: cosmetic surgeons, dermatologists, aesthetic doctors, beauty therapists. The dentist’s role in addressing the ageing face has been limited until recently, to improve the smile [15]. Recently, dentists have started to use Botox, dermal fillers and other cosmetic procedures for the face. These affect specific parts of the face, whereas the Oralift^®^ appliance is designed to address the signs of ageing on the whole face.

Standardized photography has been used to monitor the changes taking place on the face. Photography is an invaluable aid to show improvements to the patients and motivate them to persist with the prescribed regime. For this study, five patients were selected for additional 3D images in addition to the 2D format. The aim of this study was to provide measurable evidence of the changes induced by wearing the Oralift appliance. In this study, 3D imaging was used to quantify the volume changes taking place on the face. In the past, the visual assessment was limited to the appearance of the skin and this helped to formulate the regime for the patient. Could quantification of volume changes further help to formulate the regime?

## 2 MATERIALS AND METHODS

The review was undertaken on five patients treated at the clinic at 18 Wimpole Street between the dates of September 2011 and June 2012. The patients were planned to be treated at both Guy’s Dental School and the clinic. The five patients treated were friends of a patient who had already been treated. It was felt as this was a pilot study, it was not as critical for the selection procedure to be as rigid as for a randomized clinical trial.

At the first clinic visit, the patients were asked to comple te medical history forms. The only exclusion criterion was if the patient had extreme symptoms of TMD. The risks, burdens and benefits of the treatment were explained to the patients, and they were shown a series of photographs to show them the possible results of the treatment.

After a full dental examination, consent forms were explained and signed. Patients were asked to ensure that their faces were either devoid of make-up, or that their make-up was kept the same for all the visits. The patients were also weighed at the start and finish of the treatment and 2D Images were taken at the clinic. The photography was standardized by asking the patient to stand in the same position and look at the same point on the wall. They were told to keep their lips gently together without clenching their teeth. The lighting was provided by two flash units, and the settings on the camera and flash units were kept the same at each visit.

The patients were then sent to Guy’s Dental Hospital to have 3D Imaging. A standardized procedure was followed to take the images. Two sets of images were taken: one with the lips together and teeth held gently together and the other with the lips together and teeth slightly apart at a relaxed position.

Stereophotogrammetry image capture system (I Dimensional Imaging Ltd., Glasgow, Scotland, UK) was used to record an image map of the face for each of the five subjects at each visit. This involved the use of 4 linked cameras which simultaneously recorded photographs of the face. The Dimensional 3D imaging software^®^ assembled the data from the four individual cameras and converted the data into a stereolithographic format (.STL file) which stored the 3D information. Using the Dimensional imaging software^®^ the collected data could then be viewed as a 3D color photograph in 3 dimensions on the computer screen.

To measure the image difference between each patient visit, Robin’s 3D-3D Editor software (V3.1.0.0): “Robin’s Surface Scan Software”, Available from: <http://www.robins3d.co.uk>. [accessed 1 Oct 2011), was used to superimpose one image on to the other. The system of recording and overlaying images was accurate to a resolution of approximately 0.5 mm (the image capture system has an error value of < 0.2 mm).

**Fig. (2):**
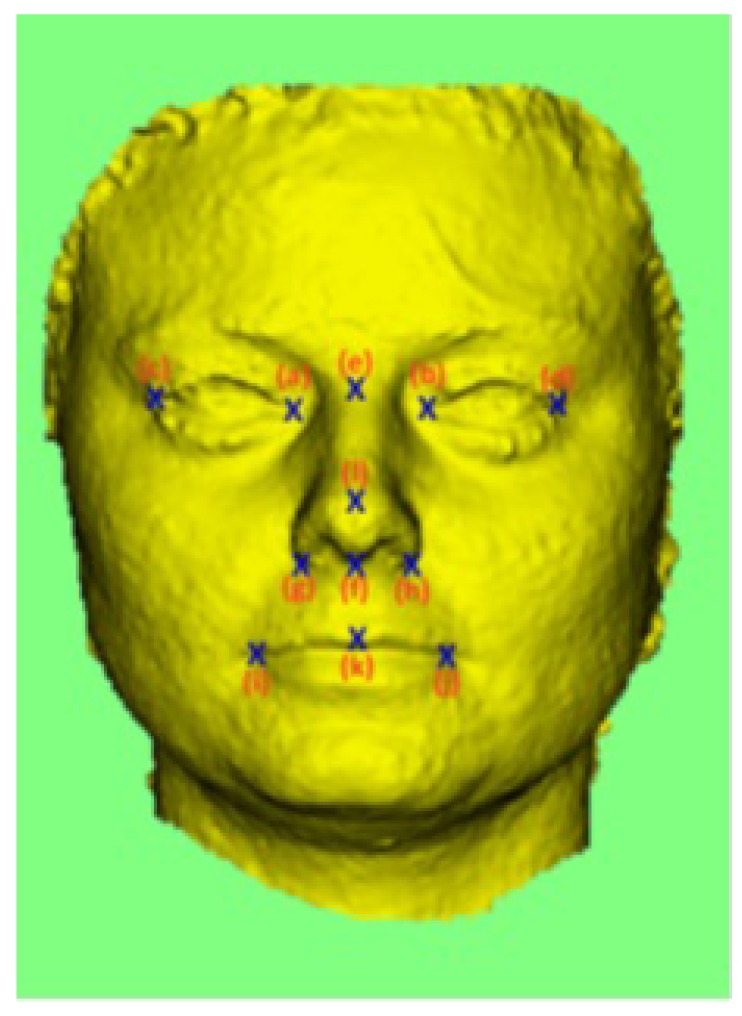
Anthropometric landmarks used in the registration process for superimposing one image onto another. (**a**) right endocanthion **b**) left endocanthion (**c**) right exocanthion (**d**) left exocanthion (**e**) nasion (**f**) subnasale (**g**) right Alare (**h**) left alare (**i**) right chelion (**j**) left chelion (**k**) labial superioris (**l**) pronasale.

The first objective is to ensure that the still image for each patient was orientated to the viewing position and saved as a view file so that the image would always return to the same x, y and z coordinates on the computer screen, making comparisons between images reproducible [16]. The same anthropometric landmarks were plotted on each image in the same order (Fig. **2**).

**Fig. (3):**
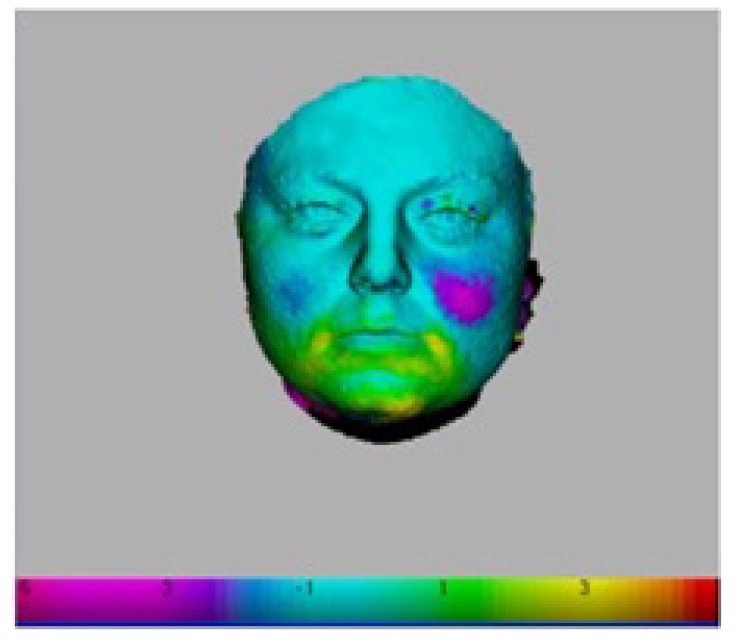
Color coding for volume changes. Pictures of patient A after 4 months of wearing the appliance.

The computer software programme then fits the two images as closely as possible, using the landmarks plotted [17]. The difference between the two surfaces is viewed as a color that was assigned a numerical value that could be set from 10.0 mm to 0.001mm. (Fig. **3**). A cursor could also be placed on the surface to confirm the difference between the surfaces.

3D volume changes were calculated with the patients’ teeth gently held together in centric occlusion and also with facial muscles relaxed and teeth not touching *i.e*. in centric relation.

At the second visit, the patients were fitted with a 3 mm and a 5 mm appliance. Before fitting the appliances it is important that the patient is made aware of their freeway space and the operator can see the movement that takes place on the face when the patient moves from the resting position to teeth held together position. The patient is first fitted with the 5 mm appliance, followed by the 3 mm appliance.

The appliance can be fitted using the “boil and bite” method or by heating it in a china bowl in a microwave. It is fitted over the mandibular teeth ensuring that there is even contact on the pivots.

After fitting each appliance, the patient has to do a test to assess the ability of the muscles to adapt to a new resting position. This test is called “The Tick Test”.

The patient is timed for five minutes and during that period, he is asked to keep his lips together and allow the jaw to rest in a new position with the 5mm appliance in the mouth. The top teeth should not touch the top of the two pivots and if they do, the patient has to put a tick on a card. The number of ticks is counted for five minutes but if the patient experiences any signs of overuse *i.e*. pain or trembling of the muscles, they are advised to stop and go on to the 3mm appliance when exactly the same procedure is followed. The patients are also taught how to swallow in a relaxed manner without the teeth touching [18]. This can be done by keeping the tip of the tongue behind the upper anterior teeth, lips together and a gentle swallowing action.

After fitting the 3mm appliance, the patient is asked to feel his freeway space again and is always surprised as how much it has increased after just 10 minutes of wearing the appliances. The operator can also see the increased movement of the face.

The regime of how to wear the appliance if no signs of overuse are experienced is shown in Table **1**.

“Lips must be kept together and teeth kept apart. If muscles start to ache or gagging is a problem, the amount of time the appliance is worn has to be reduced. Every day the appliance is worn, “the 5 minute tick test” has to be done *i.e*. a note made in a note book of the number of times the top teeth touch the top of the appliance. If the muscles are quite relaxed the teeth should touch only a few times, less than 5. If they touch more, the advice is to progress more slowly. It should be remembered that the teeth should not touch the top of the appliance even when swallowing. When swallowing, the tip of the tongue should be behind the upper 2 front teeth and the rest of the tongue will keep the back teeth apart. Some people may need to go more slowly than recommended above. Because this is going to be a lifetime regime, it is better to progress slowly. One should not move on to the higher appliance, if the muscles ache at all or if there are any signs of overuse. Muscles should never ache when the appliances are being worn.”

The patient is monitored at one month interval to ensure that the treatment is progressing satisfactorily. The criteria that need to be met are: there should be no pain from any of the facial muscles, head or neck ache, or any adverse signs of facial skin. If these criteria are not met, the patient is advised to reduce by half the amount of time wearing the appliance. At each review visit, the patient is asked if they have become more aware of their freeway space, whether they parafunction during daytime, and if they can sense a difference in the freeway space before they go to bed and when they wake up. If they have been parafunctional during the night, their freeway space is likely to be very small in the morning.

At the end of the four-month period, the patients were given two sets of questionnaires to evaluate if the treatment had any effect on their parafunctional habits and also to evaluate their perception of improvement to the facial features.

Questionnaire given to patients: (Tables **2** and **3**).

## 3 RESULTS

All five patients progressed on to the 5 mm appliance as indicated in the regime. However, one of them found that after two weeks use, the 5 mm appliance tended to make her gag and she therefore, reverted back to the 3 mm appliance.

In all the cases, improvements to the face were perceived by the patients as indicated by the answers to the questionnaire as shown in Figs. (**4** and **5**). They all thought that their skin had improved, that their cheeks had improved and that the “ageing triangle” had reversed. The ageing triangle is the downward trend that affects the lateral droop of the corners of the eyes, the corners of the mouth, flattening of the cheeks and the formation of jowls. In youth, the base of the triangle is formed by the cheeks and the chin forms the apex of the triangle. In the aged face, the base is formed by the jowls and the apex by the tip of the nose. 60% of patients reported improvement in the crows’ feet.

When answering the questionnaire about their parafunctional habits, all subjects said that they were not aware of their freeway space before treatment started. After the treatment they all confirmed that they had become aware. One patient when asked “Do you clench your teeth?” after the treatment said “No” but qualified that by commenting “ I don’t think so “. This has to be regarded that the patient was still clenching because patients must be able to answer positively if they clench their teeth during daytime. Two patients when asked: Is your FWS bigger now than before you started?, said “Yes” but added “ I think it is”. This again could not be regarded as a positive response as shown in Fig. (**6**).

When studying the changes in the volume of the face with 3D Imaging, 3 of the 5 patients showed insignificant changes in the first month when no treatment was being done. In the whole eight-month period, 3D volume changes showed that the same trends as seen in the 2D images *i.e.* more changes were apparent when the 5mm appliance was worn. The patients wore the appliances for four months continuously, followed by a four-month rest period. After the four-month of rest period, the volume changes were maintained and the face did not return to its initial state in all cases as shown in Figs. (**7** and **8**). The color coding on the 3D pictures is seen in Fig. (**3**). Some parts of the face show an increase in volume and some show a decrease in volume. 3D imaging of the patients was done with their teeth held together in centric occlusion (OVD) and also with their lips together but teeth slightly apart so that the face was in a resting position (RVD).

## 4 DISCUSSION

Earlier work has shown that changing the vertical dimension of occlusion (OVD) can have a profound effect on facial features and patients can look up to 20 years younger [19]. The ability of facial muscles and the extracellular matrix to adapt has been well documented [20, 21]. However, the facial muscles seem to adapt very quickly. The ability to adapt is gauged by the response to the “tick test”. When the initial tests are done, signs of overuse indicate very tense muscles. The purpose of the “tick test” is two- fold: first to show the patient that as the treatment progresses, the muscles start to relax and the number of ticks reduces, the second is to show the patient that tooth contact is not necessary for normal function. Tooth contact investigation has historically been a controversial subject, but modern technology will be able to demonstrate that tooth contact does not occur in normal function [22-24]. Once these two messages are embedded in the subconscious, together with the fact that the patient is starting to look better, it seems that a trigger is formed which will help to stop the patient bruxing in both day and night time. Patients have reported wakening up in the night when tooth contact occurs. The “Tick Test”, monitoring the improvements on the face with imaging, showing these improvements to the patient and making the patients aware of their parafunctional habits, form the biofeedback strategy. Biofeedback is a well-documented method to control parafunction [25-27].

The patients are also made aware of how the freeway space can change within minutes of wearing the appliance. This is the reason why, when the appliances are first fitted, it is important to fit the 5 mm appliance first followed by the 3mm appliance. While doing the tick test for the 5mm appliance, the patient’s muscles start to adapt to the new resting position and it has been observed that when the 3mm appliance is fitted, the patient finds it much easier to wear.

As shown in the results, all the patients were initially not aware of their freeway space, but by the end of treatment, they were all aware and understood the significance *i.e*. how stress or parafunctioning can reduce freeway space. However, when questioned about whether they still clench their teeth or whether their freeway space was bigger than before, two of the patients responded by saying: “I don’t think so”, indicating that although they were aware of their clenching habit and their freeway space and understood the significance, they were not fully committed to break their habit of parafunctioning.

The importance of taking pictures throughout the treatment is paramount. The improvements which can be seen, even subtle improvements, help to motivate the patients to incorporate this new regime into their lifestyle. Hitherto, only 2D images were shown to patients having the treatment and although the importance of standardization was understood, even small variations in the pictures may cause drastic changes in their clinical and research values. Areas of the face were enlarged and shown to patients to illustrate the improvements. The questionnaires which they were asked to complete, related to the areas where improvements usually take place with the Oralift treatment. The questionnaires helped to assess whether the changes shown in the 2D images were perceived and acknowledged by the patients. It was felt that 3D Imaging would do this much better because it eliminated any problems associated with change in pose and illumination. As seen in Fig. (**9**), the 3D images illustrate the improvement in crow’s feet very clearly.

Since the 3D Imaging was carried out at King’s and not in the dental clinic, the results could not always be shown to the patients in real time. The impact of the changes taking place was therefore diminished because when motivating patients, ideally changes should be shown instantaneously. 3D Imaging used in real time will overcome this problem.

3D Imaging which can show the changes in volume taking place can form an invaluable additional tool to 2D photographs. 3D Imaging provides measurable evidence that changes, however subtle, are taking place and are significant.

**Fig. (7):**
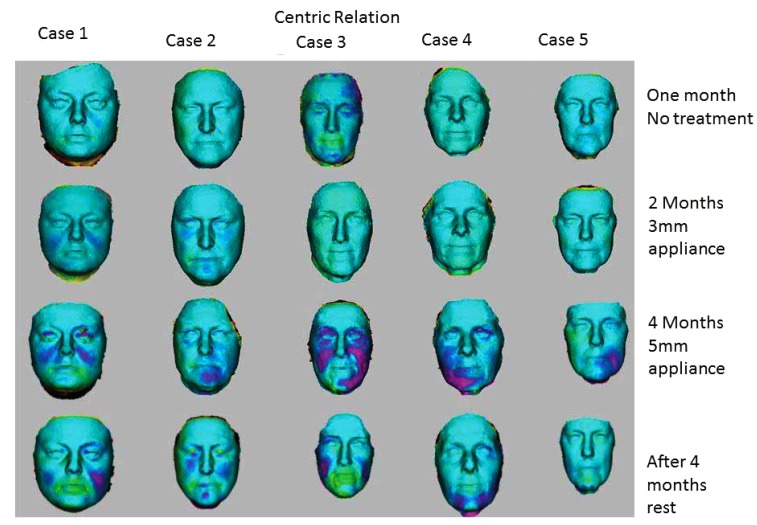
Volume changes seen in centric relation.

**Fig. (8):**
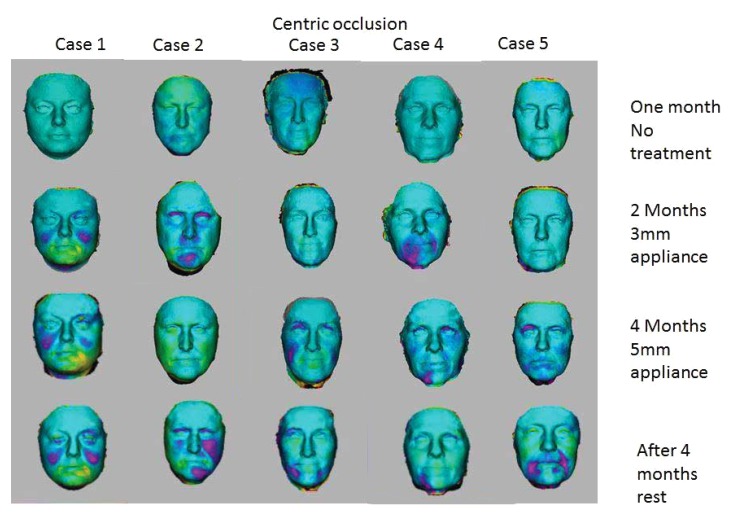
Volume changes in Centric occlusion.

The way 3D Images are superimposed on each other to measure volume changes relies on landmarks. Since the Oralift^®^ affects the whole of the face, some of the landmarks may be shifting. Despite this, the volume changes which occur when a patient wears the Oralift appliance, can be clearly seen as illustrated in (Figs. **7** and **8**).

The results of volume changes do not seem to follow a pattern. This could be attributed to the fact that the sample size is small, or that the facial muscles respond individually. A number of hypotheses could explain the volume changes seen in 3D imaging and in the changes observed in 2D imaging: correction of asymmetry, muscles trying to achieve an ideal Golden Proportion ratio, infusion of fluids into the muscles, muscle hypertrophy and change in how fat is stored in the face. The change in volume seems to be triggered by the adaptability of the muscles. When the Oralift^®^ is put in the mouth, the muscles have to adapt, and this process of adaptation may cause the muscles to alter their gene expression. This change in gene expression may activate muscle satellite stem cells which kick-start local repair [28]. It could be said that the Oralift^®^ appliance wakes up the sleeping DNA and causes a change in gene expression. It may be possible that age related changes to facial muscles are being reversed [29, 30]. If the muscles are improving, then these will affect the facial bones [31]. The bones may then start not only to strengthen but to remodel [32]. This could lead to the reversal of the bony changes that affect the face with ageing [33, 34]. All of the above need to be verified through further research.

Results with patients using this appliance vary from subtle to profound. In this sample, the results were subtle. This may be due to various factors. The ideal face follows the ratio of Golden Proportions and the more patients deviate from this ratio, it appears the more likely they are to see a more dramatic improvement [35]. Another factor which may affect the changes which are taking place is how frequently the appliance is worn. Although written instructions are given to the patient, it is not possible to ascertain exactly the number of times the appliance has been worn. One patient who was given the instructions wore the appliance every day. Another who found the 5mm appliance less comfortable wore it for shorter periods than prescribed and did not make up the time with the smaller 3 mm appliance. Parafunction may also affect the results, as would exposure to sun, diet, changes in weight, and stress.

As seen by the results, changes are maintained after the four-month rest period. When the patient starts to wear the appliance again, he is not starting from the original point. This treatment is designed to be a lifetime regime, much as physical exercise for the body has to be. The initial recommended period of wearing the appliance for four months is to train the muscles to adapt. Thereafter, the patient is recommended to wear the appliance on an ongoing basis of two months followed by four-months rest.

When we look at the answers given by the patients as to which facial features had improved, all of them stated that their skin looked better. The improvements in the skin are usually: improved texture, reduction of wrinkles, reduction in thread veins, reduction in pores, a lightening of the skin tone. However, the skin can look worse if the adaptability of the skin is exceeded. Facial muscles seem to adapt very quickly, but the skin that has lost its elasticity through the ageing process may take longer to adapt.

The changes observed are related to the process of adaptation which takes place in the facial muscles. The muscles involved seem to be not just the facial muscles but the muscles involved in breathing seem also to have to adapt to the increase in air volume in the system. The tongue has to adapt to the extra space created in the mouth, resulting in the muscles involved in swallowing having to adapt. It is likely that postural muscles which support the head on the top of the cervical vertebrae may also be affected, as the mandible attempts to find a new resting position.

Patients who come for the treatment are already motivated and want to see results. The placebo effect of coming to a clinic may already start to play a part [36]. However, when they realize that the treatment is new and no scientific trials have been done, this can have the opposite effect **i.e.** the nacebo effect [37]. Most of the work on the placebo and nocebo effects has been done in the field of pain control [38]. Nevertheless, these effects must play a role when we are looking at a treatment which is designed to be anti-ageing. It is therefore essential when doing a double blind randomized controlled study, to try to incorporate these elements into the design of the study.

When studying facial features over a long period, the weight of the patient should be accurately measured at each visit and not just at the start and the end of the four month period. Since the facial skin is being affected, the protocol for cleansing the skin and the tools necessary for measuring skin changes should be considered. When measuring volume changes, the landmarks used to superimpose images have been shifting, therefore other ways of measuring volume changes have to be considered rather than just relying on facial landmarks.

## CONCLUSION

While 3D Imaging showed the results of volume changes on the face, the outcome was that these could not be used to influence the treatment regime for the patient. A much larger study is needed to understand the trend of volume changes. 3D Imaging is very useful to monitor changes in the facial skin, but the protocol of how the imaging is done should be much stricter. 3D Imaging in these conditions could be a valuable motivational tool if done in real time. It allows very accurate standardized imaging of the face, and with further development should enable us to measure the changes very accurately. Volume changes measured by 3D imaging show that the initial results achieved by wearing the Oralift^®^ appliance are maintained even after the patient has stopped wearing the appliance for four months. This confirms the observation made with 2D imaging *i.e*. that the changes that occurred while wearing the appliance for four months did not disappear after the rest period of four months of not wearing the appliance.

New hand-held 3D cameras are now entering the market. This will facilitate taking 3D images in a clinical environment and should become part of the standard protocol for Oralift^®^ treatment.

This review of using 3D imaging of five patients has led us to the conclusion that 3D imaging can help quantify the changes taking place and should form a part of a double-blind randomized trial.

## Figures and Tables

**Fig. (4):**
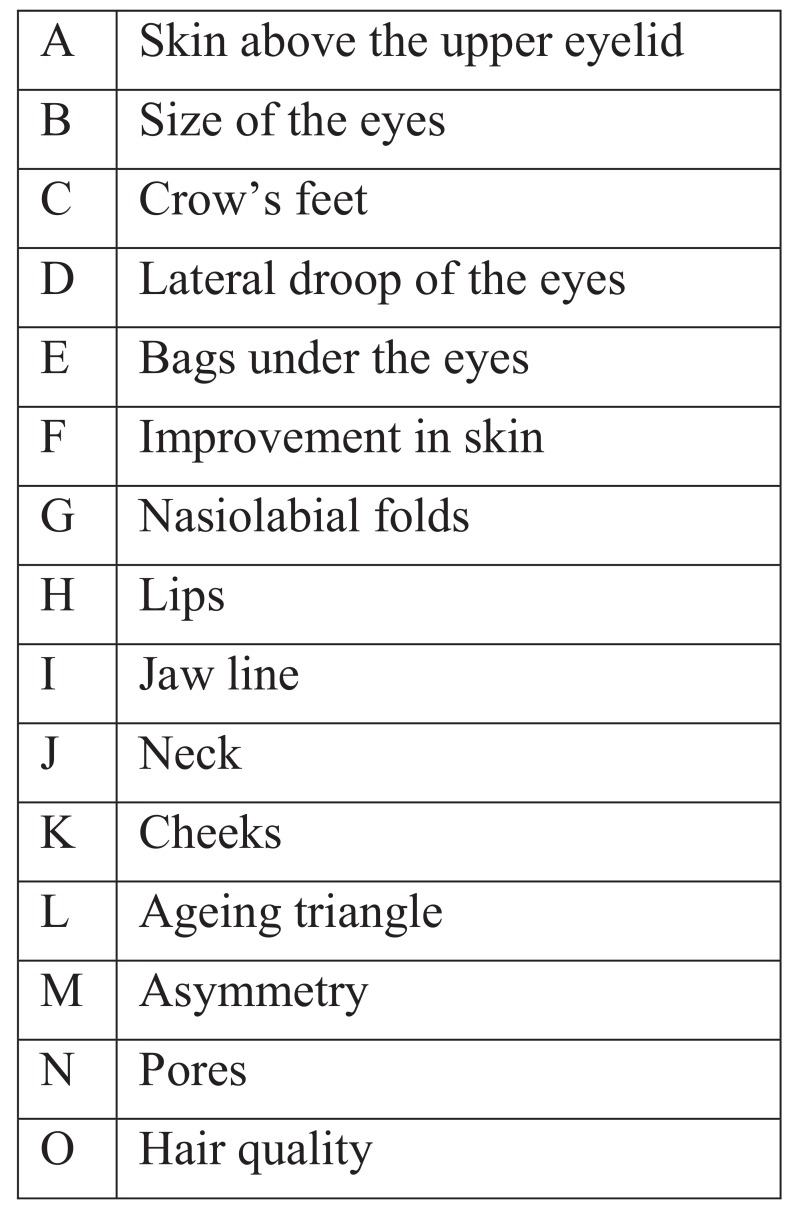
Improvements noted by patients to features of the face graded on a scale of 0-3.

**Fig. (5):**
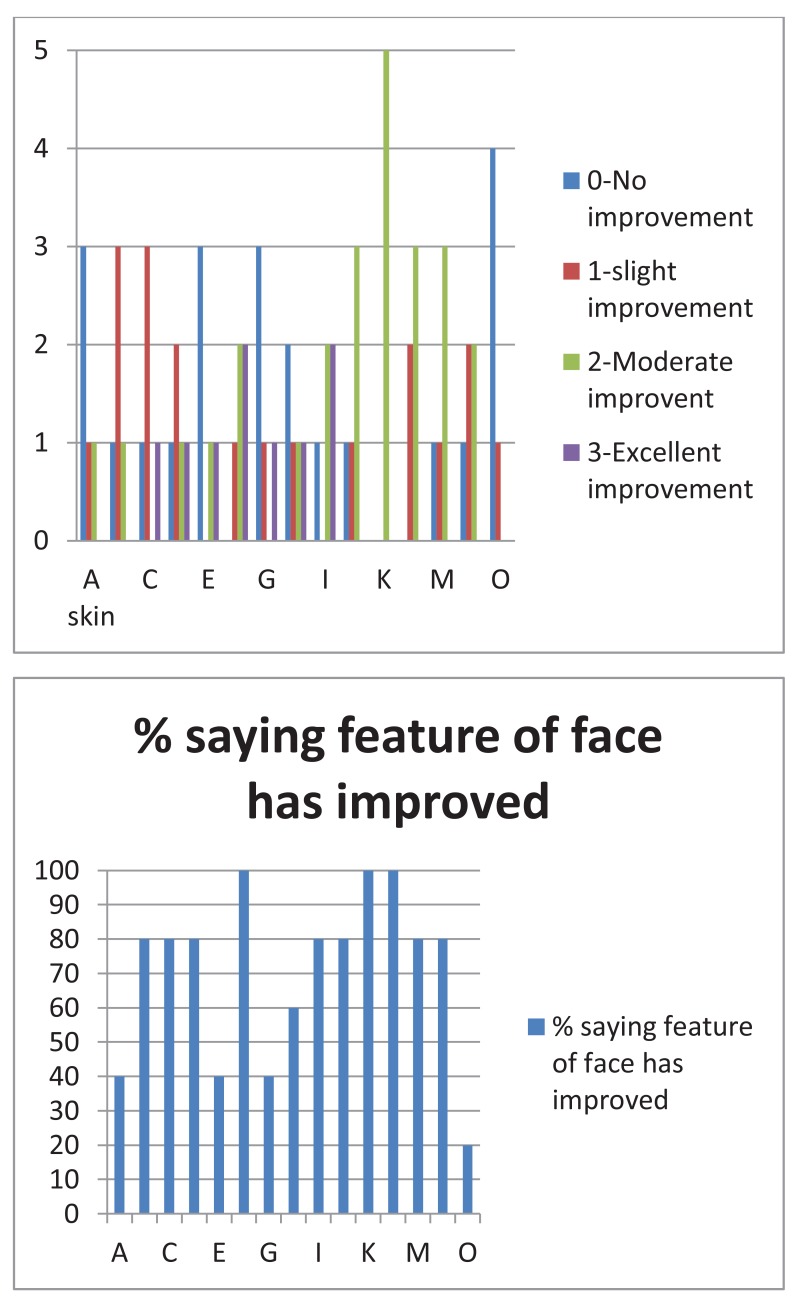
Percentage of patients who perceived an improvement in the various features of the face.

**Fig. (6):**
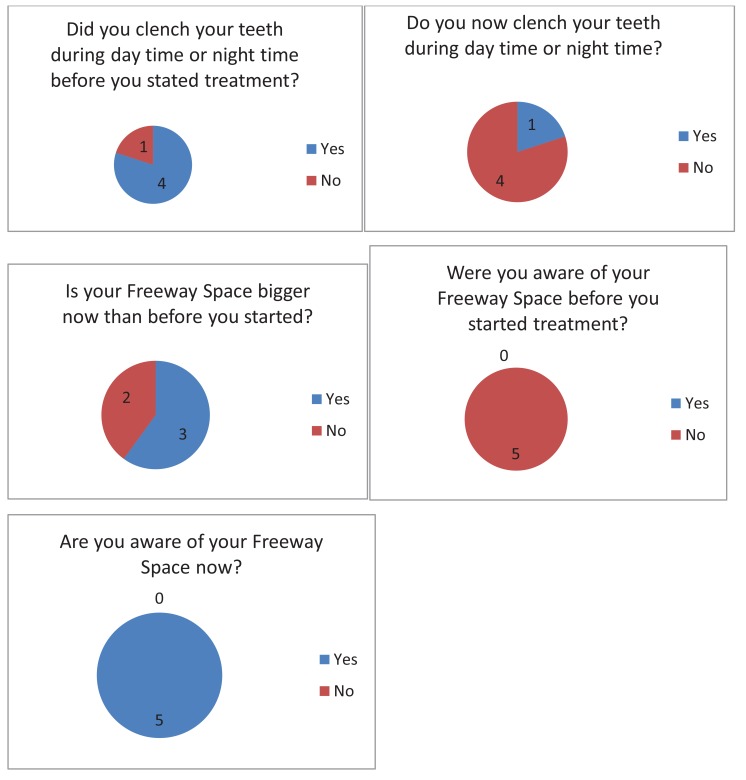
Responses to questionnaire about parafunctional habits and awareness of freeway space.

**Fig. (9):**
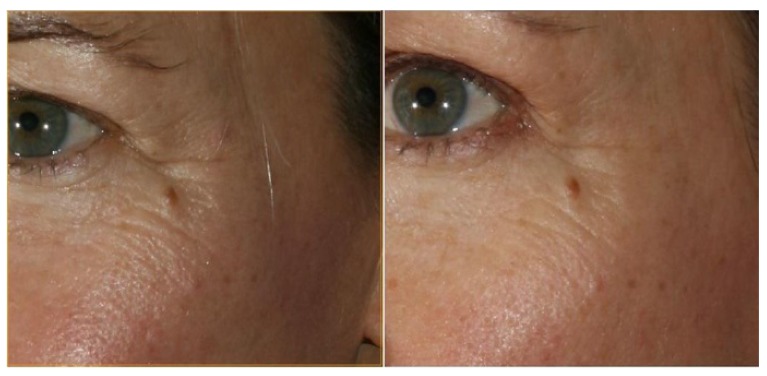
3D images showing improvement in crow’s feet after 4 months of using the Oralift appliance.

**Table1:** Regime of how to wear the Oralift^®^

3 mm Appliance	–	
Day	Time Appliance to be Worn am	Time Appliance to be Worn pm
1	15min	15min
2 & 3	Rest	Rest
4	30min	30min
5 & 6	Rest	Rest
7	45min	45min
8 & 9	Rest	Rest
10	60min	60min
11 & 12	Rest	Rest
13	Same as day 10	Same as day 10
Continue for another 4 weeks and then	–	–
Introduce 5mm appliance	–	–
61	5min with 5mm and 55min with 3mm appliance	5min with 5mm and 55min with 3mm appliance
62 & 63	Rest	Rest
64	15min with5mm and 45min with 3mm appliance	15min with 5mm and 45min with 3mm appliance
65 & 66	Rest	Rest
67	30min with 5mm and 30min with 3mm appliance	30min with 5mm and 30min with 3mm appliance
68 & 69	Rest	Rest
70	60 min with 5mm appliance	60 min with 5mm appliance
71 & 72	Rest	Rest
Continue for another 2 months with 5mm Appliance	–	–

Instructions given to patients:

**Table 2:** Questionnaire to evaluate improvement in facial features.

Which features of the face improved with the treatment? (on a scale of 0-3, 0 being none and 3 being good	–
A. skin above the eyelid	0 1 2 3
B. Size of eyes	0 1 2 3
C. Crow’s feet	0 1 2 3
D. Lateral droop of eyes	0 1 2 3
E. Bags under the eyes	0 1 2 3
F. Improvement in skin	0 1 2 3
G. Nasio-labial folds	0 1 2 3
H. Lips	0 1 2 3
I. Jaw line	0 1 2 3
J. Neck	0 1 2 3
K. Cheeks	0 1 2 3
L. Ageing triangle	0 1 2 3
M. Asymmetry	0 1 2 3
N. Pores	0 1 2 3
O. Hair Quality	0 1 2 3

**Table 3:** Questionnaire to evaluate effects on parafunctional habits and freeway space.

1. Do you clench your teeth during daytime or night time before you started your treatment?	Yes	No	Comment
2. Do you now clench your teeth during day time or night time?	Yes	No	Comment
3. Were you aware of your freeway space before you started your treatment?	Yes	No	Comment
4. Are you aware of your freeway space now?	Yes	No	Comment
5. Is your freeway space bigger now than before you started?	Yes	No	Comment
